# Longitudinal transitions of the double burden of overweight and stunting from childhood to early adulthood in India, Peru, and Vietnam

**DOI:** 10.1093/ije/dyae151

**Published:** 2024-11-14

**Authors:** Nora A Escher, Rodrigo M Carrillo-Larco, Jennie C Parnham, Katherine Curi-Quinto, Suparna Ghosh-Jerath, Christopher Millett, Paraskevi Seferidi

**Affiliations:** Public Health Policy Evaluation Unit, School of Public Health, Imperial College London, London, UK; Hubert Department of Global Health, Rollins School of Public Health, Emory University, Atlanta, GA, USA; Public Health Policy Evaluation Unit, School of Public Health, Imperial College London, London, UK; Instituto de Investigación Nutricional, Lima, Peru; Research Center of the Faculty of Health Sciences, Universidad Peruana de Ciencias Aplicadas, Lima, Peru; Department of Nutrition, The George Institute for Global Health, India; Public Health Policy Evaluation Unit, School of Public Health, Imperial College London, London, UK; NOVA National School of Public Health, Public Health Research Centre, Comprehensive Health Research Center (CHRC), NOVA University Lisbon, Lisbon, Portugal; Public Health Policy Evaluation Unit, School of Public Health, Imperial College London, London, UK

**Keywords:** Malnutrition, Markov, transition analysis, India, Peru, Vietnam

## Abstract

**Background:**

Examining trajectories of undernutrition and overnutrition separately limits understanding of the double burden of malnutrition. We investigated transitions between normal, stunting, overweight and concurrent stunting and overweight (CSO) and associations with sociodemographic factors in children and adolescents.

**Methods:**

We used data from the Young Lives cohort in India, Peru and Vietnam, which follow children 1–15 (*N* = 5413) and 8–22 years (*N* = 2225) over five rounds between 2002 and 2016. We estimated transitions between nutritional states using a Markov chain model and estimated sociodemographic associations employing a logit parametrization.

**Results:**

Transitions into stunting peaked in ages 1–5 years (India: 22.9%, Peru: 17.6%, Vietnam: 14.8%), while stunting reversal was highest during adolescence across all countries. Transitions into overweight peaked in ages 19–22, while overweight reversal increased in ages 1–5 and 12–15 years. Transitions away from stunting to overweight were rare; more commonly, stunted individuals developed overweight while remaining stunted, leading to a CSO state. In Peru, 20.2% of 19-year-olds who were stunted reached CSO by age 22, with 4% shifting from stunted to overweight. Reversion to a normal state is least likely for those in a CSO state. Household wealth gradually reduced the likelihood of transitioning into stunting [odds ratios (ORs) for wealthiest quartile in Peru: 0.29, 95% confidence interval (CI) 0.20–0.41; India: 0.43, 95% CI 0.32–0.57; Vietnam: 0.36, 95% CI 0.26–0.50), with stunting reversal only being more likely in the two wealthiest quartiles across all countries (ORs for wealthiest quartile in Peru: 2.39, 95% CI 1.57–3.65; India: 1.28, 95% CI 1.05–1.54; Vietnam: 1.89, 95% CI 1.23–2.91). In Vietnam, only the richest quartile was at higher risk of transitioning into overweight (OR 1.87, 95% CI 1.28–2.72), while in Peru and India, the risk gradually rose across all wealth quartiles (ORs for wealthiest quartile in Peru: 2.84, 95% CI 2.14–3.77; India: 2.99, 95% CI 1.61–5.54).

**Conclusions:**

Childhood and adolescence represent critical periods for prevention and reversal of stunting and overweight, thereby averting the development of CSO later in life. Context-specific interventions are crucial for preventing disparate transitions towards the double burden of malnutrition across socioeconomic groups.

Key Messages

- We analysed transitions between normal nutritional state, stunting, overweight and concurrent stunting and overweight (CSO) to understand the dynamics of the double burden of malnutrition experienced by children and adolescents in three low- and middle-income countries.
- Early childhood is pivotal for preventing stunting and overweight, while adolescence is key for reversal; CSO most commonly arises during late adolescence and early adulthood progressing primarily from stunting or, secondarily, from overweight, with limited chances of reversal.
- Understanding age-specific transition patterns is essential for designing targeted interventions to address all forms of malnutrition effectively across different life stages.

## Introduction

Addressing malnutrition is a global health priority, highlighted by Sustainable Development Goal 2.2 to eradicate all forms of malnutrition by 2030.^1^ Despite progress in reducing undernutrition, 149 million children under five were stunted in 2020, with undernutrition contributing to 45% of child deaths, primarily impacting low- and middle-income countries (LMICs).^2^ Simultaneously, due to economic progress, urbanization, and technological innovations, many LMICs are undergoing a nutrition transition, resulting in child and adolescent overweight rates increasing from 4% in 1975 to over 18% in 2016.^2–4^ Consequently, many LMICs face a double burden of malnutrition (DBM), defined as the co-occurrence of undernutrition and overnutrition within individuals, households or countries.^5^ At the individual level, the DBM can occur as concurrent stunting and overweight (CSO), affecting 1.87% of children under five globally,^5^ or as different malnutrition forms throughout life. However, lifelong DBM trajectories are poorly understood.^6^^,^^7^

Evidence suggests that different forms of malnutrition are interconnected through biological pathways.^8^ Early-life stunting is associated with inhibited fat oxidation, low energy expenditure, compromised food intake regulation, and heightened insulin resistance in later life.^9–12^ Consequently, those experiencing undernourishment in early childhood may be particularly susceptible to weight gain when exposed to obesogenic food environments.^8^ While obesity is a known risk factor for non-communicable diseases (NCDs), early-life undernutrition may exacerbate this association.^8^^,^^9^ Moreover, obese prepubertal children tend to grow faster than lean children due to heightened levels of leptin and sex hormones.^13^ However, this advantage diminishes in puberty as excess adipose tissue inhibits growth hormone secretion, increasing the risk of CSO.^13–16^

The historical focus on studying trajectories of under- and overnutrition separately limits understanding of individuals experiencing multiple forms of malnutrition simultaneously or sequentially.^17–21^ Research shows that weight and height-for-age Z-scores decline after birth, stabilizing by age 2, highlighting the importance of the first 1000 days in preventing undernutrition.^19^^,^^20^ Adolescence represents a critical period for addressing stunting through extended catch-up growth.^21^^,^^22^ Overnutrition can begin in the late fetal stage and persist throughout childhood due to exposure to obesogenic factors.^8^^,^^18^ While the body mass index (BMI) of normal-weight individuals plateaus in adolescence, that of overweight individuals stabilizes in early adulthood.^8^^,^^18^ Stunted 10-year-olds showed a higher likelihood of central fat deposition but not overall body fat 4 years later in Brazil.^23^ However, no study has investigated the simultaneous development of stunting and overweight over an extended period within the same cohort. With rapid nutrition transitions in many LMICs, we hypothesise that children and adolescents may experience different forms of malnutrition simultaneously or sequentially, influenced by sociodemographic factors. Using a multi-state model, we analyse transitions between normal nutritional state, stunting, overweight and CSO from early childhood to early adulthood and investigate associated sociodemographic variables in India, Peru and Vietnam.

## Methods

### Dataset

We used data from the Young Lives cohort in India, Ethiopia, Peru and Vietnam, collected over five rounds between 2002 and 2016.^24^ Data from Ethiopia were excluded due to low overweight prevalence (<2% across all rounds). Each country included a younger cohort (YC) with 2052 (Peru), 2011 (India) and 2000 (Vietnam) participants, and an older cohort (OC) with 714 (Peru), 1008 (India) and 1000 (Vietnam) participants.^25^ YC children were aged 1, 5, 8, 12 and 15 years across the five assessment rounds, while OC participants were aged 8, 12, 15, 19 and 22 years (±6 months).^26^ Children were randomly selected from 20 different sentinel sites in each country, with oversampling of sites in poor areas.^25^

### Anthropometric data and state definition

Height and weight were objectively measured.^27^^,^^28^ For each round, we classified participants into four nutritional states: normal, stunting, overweight and CSO. We defined stunting as height-for-age z score <−2 SD, and overweight as weight-for-height *z* score >2 SDs (<5 years), BMI-for-age >1 SD (5–19 years), and BMI >25 kg/m^2^ (>19 years), using World Health Organization (WHO) standards.^29^ We defined CSO as the co-existence of stunting and overweight in the same individual and round, while the normal state is the absence of both.

### Statistical analysis

We used R studio and the package LMest (lmestMC function).^30^ First, a discrete-time first-order four-state Markov chain model (normal, stunted, overweight and CSO) was fitted to longitudinal data without covariates (unadjusted model). The model calculates the probability distribution of all states at each time point (marginal distribution probabilities) and estimates probabilities of moving between states over time (transition probabilities). We assumed time-inhomogeneity of transitions, where the model estimates unique transition probabilities for each interval (lmestMC option: modBasic = 0). For the unadjusted four-state model, we restricted the sample to children present in all rounds with complete anthropometric data using listwise deletion (Supplementary Table S1, available as Supplementary data at *IJE* online). We compared baseline sociodemographic characteristics for included and excluded participants (Supplementary Table S2, available as Supplementary data at *IJE* online). Separate models were fitted for each country.

Second, we investigated the association between covariates and transitions in and out of each malnourished state using three distinct two-state Markov chain models: stunted/not stunted, overweight/not overweight and CSO/not CSO. Each model utilized a binary logit parameterization to incorporate time-varying covariates (adjusted models). This approach estimates how covariates influence the likelihood of transitioning between states compared with staying in a reference state, expressed as odds ratios (OR). Each OR quantifies the effect of a unit change in the covariate on the probability of transitioning between states compared with remaining in the original state.^30^ We used listwise deletion to handle missing covariate data (Supplementary Table S1, available as Supplementary data at *IJE* online).

Covariates for the two-state models were selected based on literature from the included countries, ensuring none of them is on the causal pathway between other covariates and stunting or overweight, and univariate logistic regressions with stunting and overweight at baseline. Variables with a significant association (*P *<* *0.05) with either outcome in at least one country were considered. Final model selection was based on the Akaike information criterion (AIC) due to its suitability in smaller samples and less penalization of additional parameters (Supplementary Table S4, available as Supplementary data at *IJE* online).^31^ This involved comparing univariate models and models with stepwise covariate additions. Wealth index (quartiles),^32^ sex (male/female), place of residence (urban/rural) and household size (≤5 members/>5 members) were selected, as they resulted in the lowest AIC in most of the two-state models. Although some models with small sample size (CSO models) showed a better fit for unadjusted or univariate models, we present the fully adjusted model for consistency. Estimates from univariate models are presented in Supplementary Table S5, available as Supplementary data at *IJE* online. We compared the direction of effect of the univariate and the fully adjusted models (Supplementary Table S6, available as Supplementary data at *IJE* online) and found it consistent. Despite showing an association with stunting and overweight at baseline, maternal education was excluded due to a high proportion of missing data (up to 30%).

### Sensitivity analysis

As growth patterns between boys and girls vary, we estimated transition probability separately by sex. Additionally, we ran a separate model adjusting for maternal education (Supplementary Table S1, available as Supplementary data at *IJE* online).

## Results

### Study participants

The final sample size for the unadjusted four-state model was 5413 (89%) for YC and 2225 (82%) for OC. For the adjusted two-state models, it was 5278 (87%) for YC and 2129 (78%) for OC (Supplementary Table S1, available as Supplementary data at *IJE* online). Table 1 displays baseline characteristics by nutritional state.

**Table 1.** Baseline characteristics for children from India, Peru and Vietnam by their baseline nutritional status for participants included in final model (adjusted four-state model)

India YC		Normal (*N* = 1331, 74.5%)	Stunted (*N* = 448, 25.1%)	Overweight (*N* = 4, 0.2%)	CSO (*N* = 4, 0.2%)	Total (*N* = 1787, 100%)	*P*
Sex	Male	699 (52.5%)	263 (58.7%)	3 (75.0%)	2 (50.0%)	967 (54.1%)	0.117
	Female	632 (47.5%)	185 (41.3%)	1 (25.0%)	2 (50.0%)	820 (45.9%)	
Wealth	Q1	290 (21.8%)	164 (36.6%)	0 (0%)	2 (50.0%)	456 (25.5%)	<0.001
	Q2	332 (24.9%)	116 (25.9%)	1 (25.0%)	1 (25.0%)	450 (25.2%)	
	Q3	347 (26.1%)	101 (22.5%)	2 (50.0%)	0 (0%)	450 (25.2%)	
	Q4	362 (27.2%)	67 (15.0%)	1 (25.0%)	1 (25.0%)	431 (24.1%)	
Residence	Urban	363 (27.3%)	70 (15.6%)	2 (50.0%)	1 (25.0%)	436 (24.4%)	<0.001
	Rural	968 (72.7%)	378 (84.4%)	2 (50.0%)	3 (75.0%)	1351 (75.6%)	
Household size	≤5 members	817 (61.4%)	265 (59.2%)	3 (75.0%)	4 (100.0%)	1089 (60.9%)	0.307
	>5 members	514 (38.6%)	183 (40.8%)	1 (25.0%)	0 (0%)	698 (39.1%)	
Maternal education	≤6 years	969 (73.0%)	372 (83.4%)	2 (50.0%)	3 (100.0%)	1346 (75.6%)	<0.001
	>6 years	359 (27.0%)	74 (16.6%)	2 (50.0%)	0 (0%)	435 (24.4%)	

India OC		Normal (*N* = 576, 67.4%)	Stunted (*N* = 268, 31.4%)	Overweight (*N* = 9, 1.1%)	CSO (*N* = 2, 0.2%)	Total (*N* = 855, 100%)	*P*
Sex	Male	285 (49.5%)	137 (51.1%)	6 (66.7%)	0 (0%)	428 (50.1%)	0.362
	Female	291 (50.5%)	131 (48.9%)	3 (33.3%)	2 (100.0%)	427 (49.9%)	
Wealth	Q1	136 (23.6%)	75 (28.0%)	2 (22.2%)	1 (50.0%)	214 (25.0%)	0.010
	Q2	138 (24.0%)	78 (29.1%)	0 (0%)	0 (0%)	216 (25.3%)	
	Q3	143 (24.8%)	67 (25.0%)	1 (11.1%)	1 (50.0%)	212 (24.8%)	
	Q4	159 (27.6%)	48 (17.9%)	6 (66.7%)	0 (0%)	213 (24.9%)	
Residence	Urban	149 (25.9%)	41 (15.3%)	6 (66.7%)	0 (0%)	196 (22.9%)	<0.001
	Rural	427 (74.1%)	227 (84.7%)	3 (33.3%)	2 (100.0%)	659 (77.1%)	
Household size	≤5 members	365 (63.4%)	157 (58.6%)	5 (55.6%)	0 (0%)	527 (61.6%)	0.162
	>5 members	211 (36.6%)	111 (41.4%)	4 (44.4%)	2 (100.0%)	328 (38.4%)	
Maternal education	≤6 years	458 (80.2%)	236 (90.1%)	4 (44.4%)	2 (100.0%)	700 (82.9%)	<0.001
	>6 years	113 (19.8%)	26 (9.9%)	5 (55.6%)	0 (0%)	144 (17.1%)	

Peru YC		Normal (*N* = 1210, 70.1%)	Stunted (*N* = 330, 19.1%)	Overweight (*N* = 151, 8.6%)	CSO (*N* = 34, 2.0%)	Total (*N* = 1725, 100%)	*P*
Sex	Male	583 (48.2%)	195 (59.1%)	74 (49.0%)	19 (55.9%)	871 (50.5%)	0.005
	Female	627 (51.8%)	135 (40.9%)	77 (51.0%)	15 (44.1%)	854 (49.5%)	
Wealth	Q1	275 (22.7%)	127 (38.5%)	19 (12.6%)	11 (32.4%)	432 (25.0%)	<0.001
	Q2	280 (23.1%)	125 (37.9%)	20 (13.3%)	7 (20.6%)	432 (25.0%)	
	Q3	332 (27.4%)	46 (13.9%)	43 (28.5%)	11 (32.4%)	432 (25.0%)	
	Q4	323 (26.7%)	32 (9.7%)	69 (45.7%)	5 (14.7%)	429 (24.9%)	
Residence	Urban	880 (72.7%)	157 (47.6%)	131 (86.6%)	22 (64.7%)	1190 (69.0%)	<0.001
	Rural	330 (27.3%)	173 (52.4%)	20 (13.3%)	12 (35.3%)	535 (31.0%)	
Household size	≤5 members	673 (55.6%)	148 (44.8%)	84 (55.6%)	17 (50.0%)	922 (53.5%)	0.006
	>5 members	537 (44.4%)	182 (55.2%)	67 (44.4%)	17 (50.0%)	803 (46.5%)	
Maternal education	≤6 years	518 (43.7%)	236 (73.5%)	49 (33.1%)	19 (61.3%)	822 (48.8%)	<0.001
	>6 years	667 (56.3%)	85 (26.5%)	99 (66.9%)	12 (38.7%)	863 (51.2%)	

Peru OC		Normal (*N* = 292, 55.0%)	Stunted (*N* = 104, 19.6%)	Overweight (*N* = 102, 19.2%)	CSO (*N* = 33, 6.2%)	Total (*N* = 531, 100%)	*P*
Sex	Male	143 (49.0%)	53 (51.0%)	55 (53.9%)	23 (69.7%)	274 (51.6%)	0.147
	Female	149 (51.0%)	51 (49.0%)	47 (46.1%)	10 (30.3%)	257 (48.4%)	
Wealth	Q1	61 (20.9%)	49 (47.1%)	14 (13.7%)	9 (27.3%)	133 (25.1%)	<0.001
	Q2	76 (26.0%)	30 (28.9%)	20 (19.6%)	9 (27.3%)	135 (25.4%)	
	Q3	76 (26.0%)	14 (13.5%)	34 (33.3%)	8 (24.2%)	132 (24.9%)	
	Q4	79 (27.1%)	11 (10.6%)	34 (33.3%)	7 (21.2%)	131 (24.7%)	
Residence	Urban	229 (78.4%)	60 (57.7%)	91 (89.2%)	24 (72.7%)	404 (76.1%)	<0.001
	Rural	63 (21.6%)	44 (42.3%)	11 (10.8%)	9 (27.3%)	127 (23.9%)	
Household size	≤5 members	170 (58.2%)	40 (38.5%)	71 (69.6%)	16 (48.5%)	297 (55.9%)	<0.001
	>5 members	122 (41.8%)	64 (61.5%)	31 (30.4%)	17 (51.5%)	234 (44.1%)	
Maternal education	≤6 years	121 (42.9%)	67 (68.4%)	44 (45.4%)	16 (51.6%)	248 (48.8%)	<0.001
	>6 years	161 (57.1%)	31 (31.6%)	53 (54.6%)	15 (48.4%)	260 (51.2%)	

Vietnam YC		Normal (*N* = 1500, 84.9%)	Stunted (*N* = 246, 13.9%)	Overweight (*N* = 16, 0.9%)	CSO (*N* = 4, 0.23%)	Total (*N* = 1766, 100%)	*P*
Sex	Male	750 (50.0%)	145 (58.9%)	12 (75.0%)	2 (50.0%)	909 (51.5%)	0.014
	Female	750 (50.0%)	101 (41.1%)	4 (25.0%)	2 (50.0%)	857 (48.5%)	
Wealth	Q1	339 (22.6%)	102 (41.5%)	2 (12.5%)	2 (50.0%)	445 (25.2%)	<0.001
	Q2	373 (24.9%)	62 (25.2%)	2 (12.5%)	1 (25.0%)	438 (24.8%)	
	Q3	389 (25.9%)	62 (25.2%)	0 (0%)	1 (25.0%)	452 (25.6%)	
	Q4	399 (26.6%)	20 (8.1%)	12 (75.0%)	0 (0%)	431 (24.4%)	
Residence	Urban	287 (19.1%)	17 (6.9%)	8 (50.0%)	0 (0%)	312 (17.7%)	<0.001
	Rural	1213 (80.9%)	229 (93.1%)	8 (50.0%)	4 (100.0%)	1454 (82.3%)	
Household size	≤5 members	1069 (71.3%)	173 (70.3%)	9 (56.3%)	2 (50.0%)	1253 (70.9%)	0.456
	>5 members	431 (28.7%)	73 (29.7%)	7 (43.7%)	2 (50.0%)	513 (29.1%)	
Maternal education	≤6 years	802 (53.9%)	169 (68.7%)	4 (25.0%)	4 (100.0%)	979 (55.8%)	<0.001
	>6 years	686 (46.1%)	77 (31.3%)	12 (75.0%)	0 (0%)	775 (44.2%)	

Vietnam OC		Normal (*N* = 552, 70.3%)	Stunted (*N* = 202, 27.2%)	Overweight (*N* = 16, 2.2%)	CSO (*N* = 3, 0.4%)	Total (*N* = 743, 100%)	*P*
Sex	Male	235 (45.0%)	116 (57.4%)	13 (81.3%)	2 (66.7%)	366 (49.3%)	0.001
	Female	287 (55.0%)	86 (42.6%)	3 (18.8%)	1 (33.3%)	377 (50.7%)	
Wealth	Q1	124 (23.8%)	57 (28.2%)	2 (12.5%)	3 (100.0%)	186 (25.0%)	<0.001
	Q2	129 (24.7%)	65 (32.2%)	1 (6.3%)	0 (0%)	195 (26.2%)	
	Q3	135 (25.9%)	47 (23.3%)	3 (18.8%)	0 (0%)	185 (24.9%)	
	Q4	134 (25.7%)	33 (16.3%)	10 (62.5%)	0 (0%)	177 (23.8%)	
Residence	Urban	102 (19.5%)	31 (15.4%)	9 (56.3%)	0 (0%)	142 (19.1%)	0.001
	Rural	420 (80.5%)	171 (84.7%)	7 (43.8%)	3 (100.0%)	601 (80.9%)	
Household size	≤5 members	404 (77.4%)	136 (67.3%)	14 (87.5%)	0 (0%)	554 (74.6%)	<0.001
	>5 members	118 (22.6%)	66 (32.7%)	2 (12.5%)	3 (100.0%)	189 (25.4%)	
Maternal education	≤6 years	254 (49.1%)	132 (67.7%)	6 (37.5%)	3 (100.0%)	395 (54.4%)	<0.001
	>6 years	263 (50.9%)	63 (32.3%)	10 (62.5%)	0 (0%)	336 (46.0%)	

CSO, concurrent stunting and overweight; OC, Older Cohort; YC, Younger Cohort.

Wealth levels indicate quartiles of a wealth index (Q1 being the poorest). *P*-values were calculated using chi-squared tests, assessing associations across columns.

Normal state prevalence ranged from 55.0% in Peru’s OC to 84.9% in Vietnam’s YC. Among malnourished states, stunting had the highest baseline prevalence in all countries, ranging from 13.9% in Vietnam's YC to 31.4% in India's OC. In India and Vietnam, overweight and CSO at baseline were <2.5%, while in Peru, overweight was 19.2% and CSO 6.2% (OC).

### Marginal distribution probabilities of states

**Figure 1.**
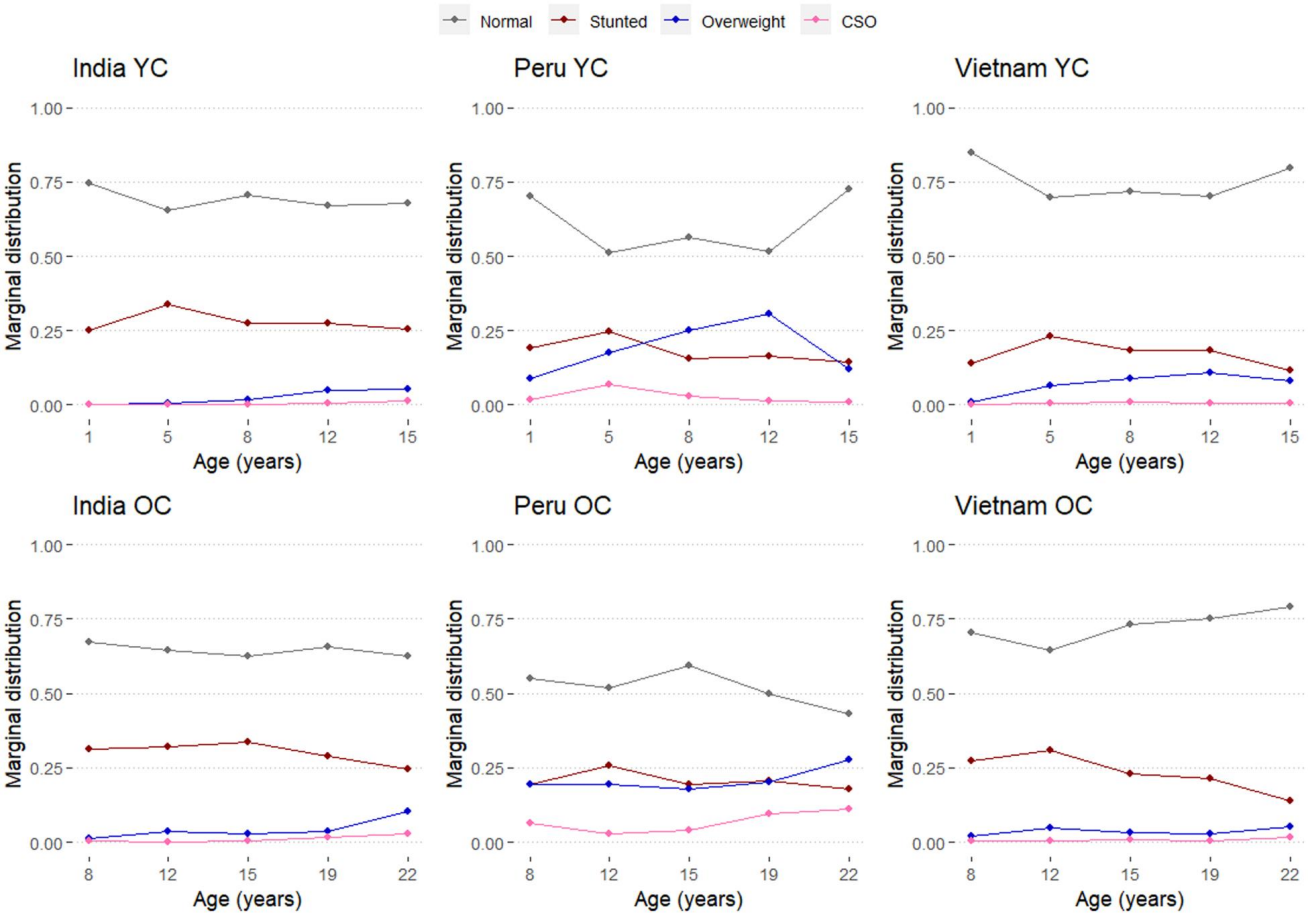
Marginal distribution probabilities of normal, stunting, overweight and concurrently stunted and overweight (CSO) for children aged 1–15 [younger cohort (YC)] and 8–22 [older cohort (OC)] from unadjusted four-state models

Figure 1 displays marginal distribution probabilities from the unadjusted four-state model. Stunting peaked among 5-year-olds across all countries (India: 33.7%, Peru: 24.6%, Vietnam: 23.1%). In India and Vietnam, stunting and overweight rates converged, with overweight nearly reaching stunting prevalence in Vietnam’s YC at age 15 (8.1% overweight, 11.7% stunting). In Peru, overweight surpassed stunting at times, exceeding 25% among 12- and 22-year-olds. CSO prevalence remained low in India and Vietnam (<2%), but steadily increased in Peru, reaching 10.1% among 22-year-olds (OC).

### Transitions between states

**Figure 2.**
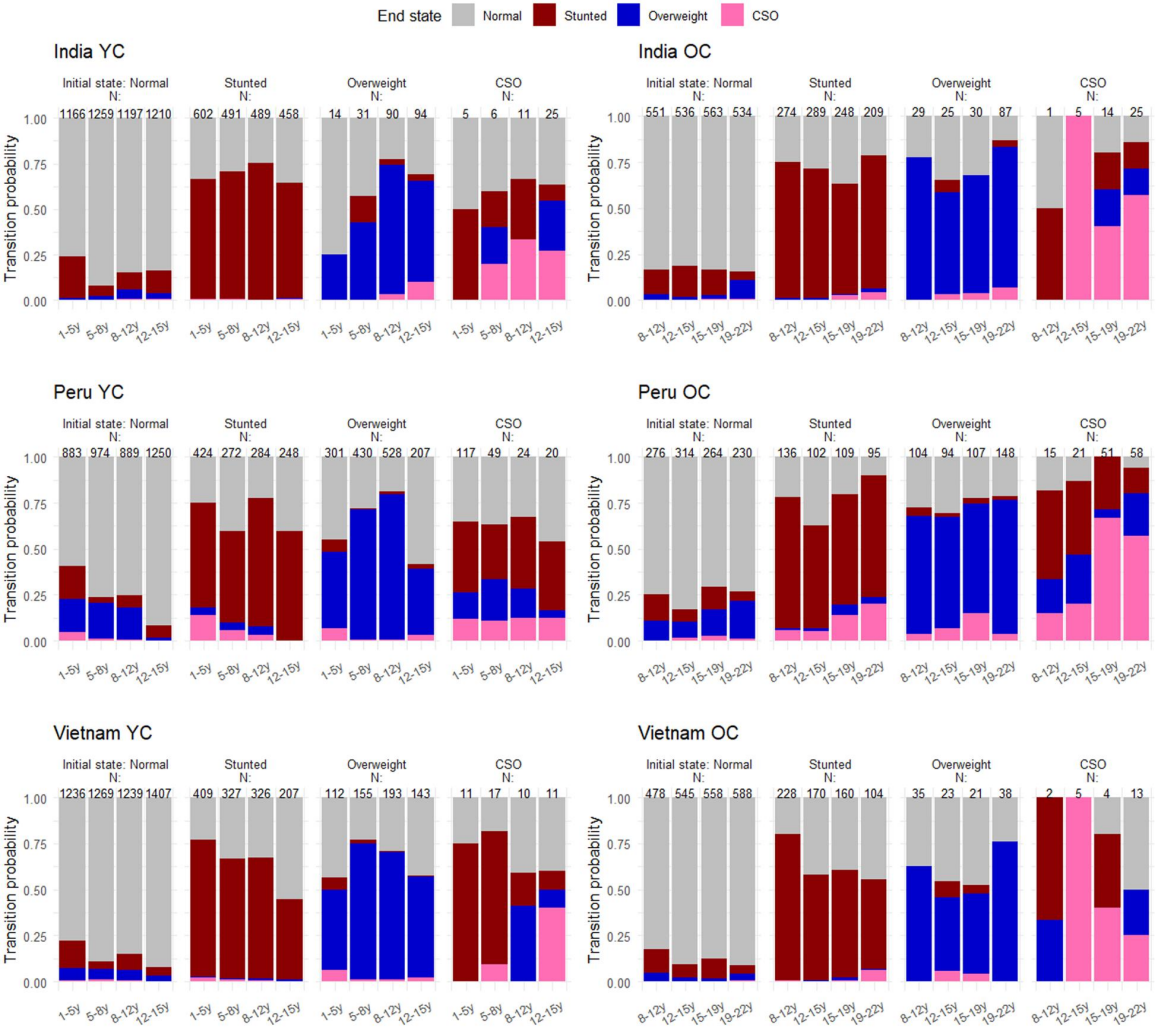
Transition probabilities between normal, stunted, overweight and concurrently stunted and overweight (CSO) state over time for the younger cohort (YC) and older cohort (OC) from unadjusted four-state models. Initial states are indicated in the columns and end states are indicated by colours. Transition probabilities estimates and SEs are shown in Supplementary Table S3, available as Supplementary data at *IJE* online

Figure 2 shows the transition probabilities from the unadjusted four-state model, and estimates and SE are shown in Supplementary Table 3, available as Supplementary data at *IJE* online. Despite varying magnitudes of malnourished states across countries, similar temporal patterns in transitions emerged. Children were more likely to remain in their original nutritional state than to transition. The highest likelihood of transitioning from normal to stunted occurred between 1 and 5 years, with rates of 22.9% ± 1.2 (SE), 17.6% ± 1.1 (SE) and 14.8% ± 0.9 (SE) for India, Peru and Vietnam, respectively. Transitions from stunting to normal state increased between 12 and 15 years in OC and YC, ranging from 28.5% ± 2.7 (SE) in India’s OC to 55.7% ± 2.8 (SE) in Vietnam’s YC.

In India and Peru, transitions from normal to overweight were most likely between 19 and 22 years [9.9% ± 1.3 (SE) and 20.5% ± 2.5 (SE), respectively]. In Vietnam, transition probabilities into overweight were low. Overweight children mostly reverted to a normal state between 1 and 5 years [India: 75.0% ± 21.7 (SE), Peru: 45.0% ± 4.5 (SE), Vietnam: 43.8% ± 12.4 (SE)], with another peak between ages 12 and 15. After age 19, transitioning from overweight to normal reached a minimum in all countries (<25%).

Few children transitioned from stunting to overweight, or vice versa. More frequently, stunted children developed overweight while remaining stunted, leading to CSO. In Peru’s OC, the trend of stunted children becoming CSO increased over time, with 13.7% ± 3.4 (SE) at ages 15–19 and 20.2% ± 3.8 (SE) at ages 19–22. Transitions from stunted to CSO were rare in India and Vietnam but peaked between 19 and 22 [India: 4.0% ± 1.2 (SE), Vietnam: 6.3% ± 1.9 (SE)]. Transitions into CSO also arose frequently among overweight adolescents, with the highest probabilities among Peru’s 15- to 19-year-olds [14.9% ± 3.7 (SE)] and India’s 12- to 15-year-olds (YC, 10.0% ± 3.2). Likelihood of recovering from CSO decreased with age, although small CSO prevalence (N < 100) limits robust conclusions.

### Associations between covariates and transitions

**Figure 3.**
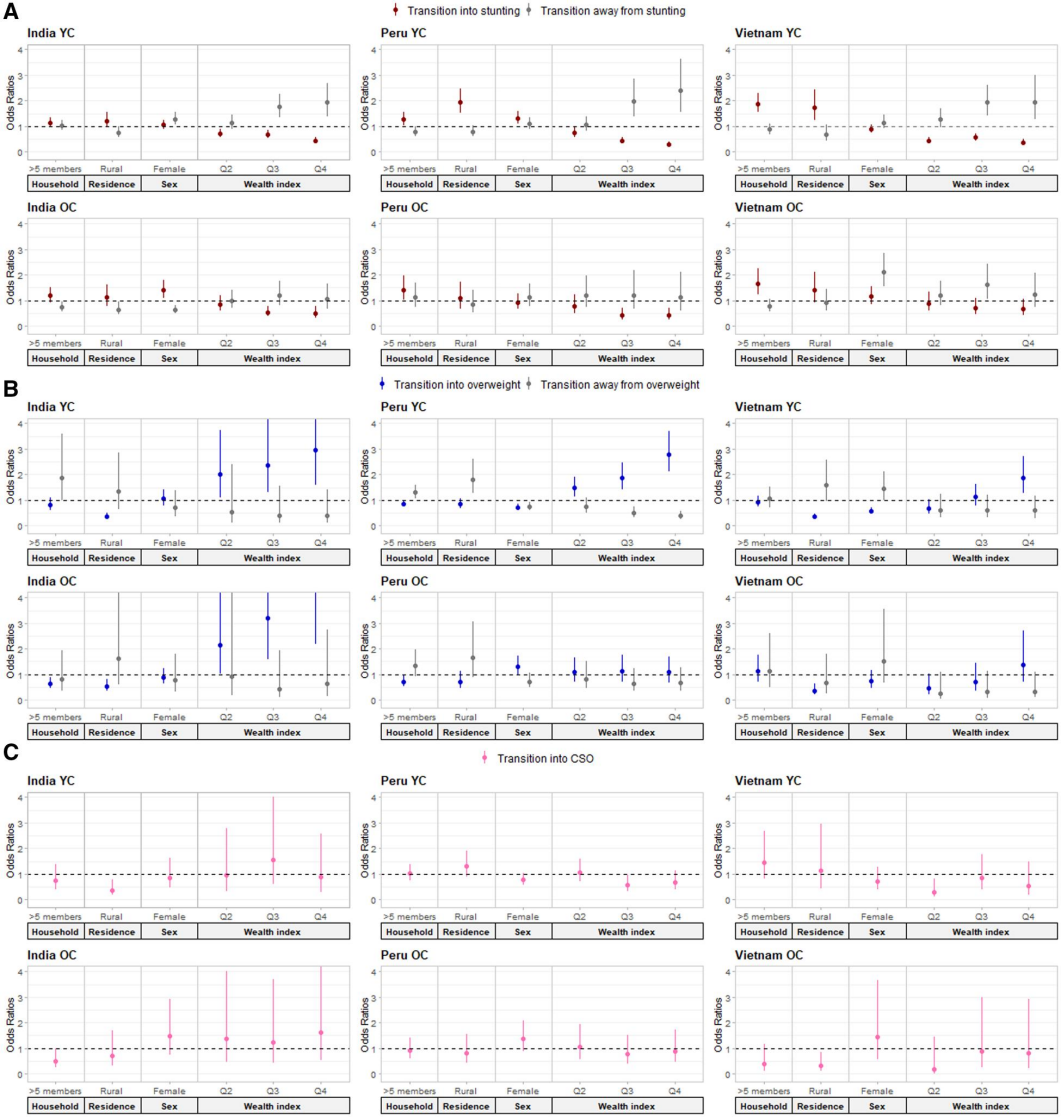
Odds ratios (OR) and 95% confidence interval (CI) showing the relative difference for transitions into and out of stunting (A), overweight (B), and concurrent stunting and overweight (CSO, C) by sex, household size, residence, and wealth quartile from adjusted two-state models. No estimates are shown for transitions out of CSO due to small sample size. OR and 95% CI estimates are shown in Supplementary Table S6, available as Supplementary data at *IJE* online

Figure 3 shows associations between covariates and transitions in and out of malnourished states using adjusted two-state models. Higher household wealth consistently lowered the chance of becoming stunted, with positive associations between wealth and stunting recovery observed in the upper two wealth quartiles of YCs (Supplementary Table S6, available as Supplementary data at *IJE* online). Wealth incrementally increased the likelihood of transitioning into overweight in India (YC and OC) and Peru (YC only), while in Vietnam, this was seen only in the YC’s richest quartile (OR 1.89, 95% CI 1.23–2.91). Rural residency increased transitions into stunting in the YC of Peru (OR 1.90, 95% CI 1.49–2.42) and Vietnam (OR 1.71, 95% CI 1.21–2.40), and lowered chances of transitioning out of stunting in India (YC and OC). Rural residents in India and Vietnam had lower risk of transitioning into overweight compared with urban residents in both cohorts and lower risk of transitioning into CSO in India’s YC (OR 0.38, 95% CI 0.18–0.80) and Vietnam’s OC (OR 0.30, 95% CI 0.11–0.84). Factors associated with transitioning away from CSO could not be reliably calculated due to the small number of individuals experiencing this transition (Figure 3C).

### Sensitivity analyses

We examined growth patterns by sex (YC only). Both sexes showed similar marginal distributions (Supplementary Figure S1, available as Supplementary data at *IJE* online) and transition probabilities (Supplementary Figure S2, available as Supplementary data at *IJE* online). Adjusting for maternal education attenuated the link between wealth and transitions into stunting in Peru and Vietnam’s OC, but no other associations were significantly affected.

## Discussion

Our study investigated transitions between normal nutritional state, stunting, overweight and CSO and determinants of transitions across three LMICs from early childhood to young adulthood. The highest likelihood of transitioning into stunting occurred between 1 and 5 years, while adolescence appeared favourable for reverting stunting. Transitions from overweight to normal state were lowest after age 19 in all countries. The probability of transitioning into CSO was higher for those already stunted or overweight. Household wealth and urban residence decreased the likelihood of transitioning into stunting, with higher recovery observed among the upper two wealth quartiles. The association between wealth and transitions into overweight varied across countries, reflecting different stages in countries’ nutrition transition.

Our findings highlight developmental periods crucial for tackling undernutrition and overnutrition. Transitioning into stunting was most likely before age 5, aligning with the window of opportunity for preventing undernutrition in early childhood.^19^ Transitions from stunting to normal were most likely between ages 12 and 15, supporting the second window of opportunity against stunting through growth in adolescence.^17^^,^^21^ The likelihood of transitioning from overweight to normal state showed a decreasing trend with age but increased notably between ages 12 and 15, before reaching a minimum between ages 19 and 22. This peak may be attributed to the elevated basal metabolic rate during adolescence, driven by growth and puberty-related hormones.^33^^,^^34^ Previous research found that <1% of obese adults achieve normal weight over a 10-year follow-up, highlighting the opportunity prevention and reversal during adolescence before physical plasticity diminishes in adulthood.^35^^,^^36^

The Markov chain enables examination of transitions between nutritional states over time, capturing the simultaneous and sequential co-existence of stunting and overweight.^17^ Existing epidemiological research on CSO focuses on prevalence and cross-sectional determinants, particularly in children under 5.^37^^,^^38^ Our study reveals a sequential progression into CSO, originating from stunting or overweight. Transitions from stunting to CSO were most frequent in Peru, with over 20% of all stunted children at age 19 becoming CSO by age 22. Metabolic consequences related to stunting, such as impaired fat oxidation, may increase susceptibility to high-fat diets, potentially explaining increased fat accumulation among stunted individuals in countries undergoing nutrition transition.^8^^,^^9^ We observed transitions from overweight into CSO, mainly in Peruvian and Indian adolescents. Research shows that while overweight individuals are taller than their normal-weight peers during early childhood, this advantage diminishes in adolescence due to suppressed growth hormone secretion in children with excess adipose tissue, potentially leading to a growth backlog.^13–16^ These findings underscore the importance of early measures for both stunting and overweight to prevent CSO, mitigating long-term consequences associated with this condition.

We examined countries at distinct stages of nutrition transition, reflecting different levels of economic development.^4^ India and Vietnam, both lower-middle-income countries, experienced declining stunting and rising overweight, yet overweight remained mostly below 10% and CSO low.^39^ Previous research suggests that as overweight prevalence increases up to 10%, stunting decreases and CSO remains low, but above 10% overweight prevalence, stunting stops decreasing, and CSO rises.^40^ In Peru, an upper-middle-income country, overweight exceeded 25%, surpassing stunting, while CSO rose above 10%, illustrating the consequences of an advanced nutrition transition. Our findings suggest that lower socioeconomic groups are at higher risk of DBM as nutrition transition advances. Across countries, transitions away from stunting were more likely in the two wealthiest quartiles and urban areas, highlighting poverty-related stunting persistence and urban–rural disparities.^17^^,^^41^ In Vietnam, transitions into overweight were more likely in the wealthiest quartile, while in India and Peru, the risk gradually rose across all wealth quartiles. This mirrors the early to intermediate stages of nutrition transition, where obesity initially emerges among the most affluent and then shifts to less affluent groups. Previous research in Peru revealed a shift from healthy to unhealthy food consumption across all social groups, potentially contributing to rising overweight among less affluent and rural populations.^42^ As economies and nutrition transition progress, context-specific interventions are crucial to address the growing burden of CSO and alleviate disparate transitions towards the DBM.^43^

We identify several policy implications. First, given the risk of individual-level DBM in countries undergoing nutrition transition, it is imperative to integrate double-duty targets into nutrition interventions to ensure that efforts to address undernutrition do not inadvertently worsen overweight.^44^ Second, the high transition probabilities in early childhood highlight the importance of maternal and child health programmes against the DBM. Third, interventions during adolescence can offer dual benefits due to high stunting and overweight reversal, while establishing healthy habits early on.^45^ Fourth, recognizing the reduced likelihood of stunting recovery among the poorest, income-based policies can help reduce stunting inequalities, provided that healthy food environments are protected to prevent spending extra income on unhealthy food.^46^

This study has numerous strengths. First, using a Markov chain allowed us to capture transitions in and out of malnourished states along with associated covariates. By incorporating a CSO state, we explore a dimension not previously examined longitudinally, offering insights into the individual-level DBM over the first two decades of life across three countries at different economic stages. Second, despite the study's 15-year duration, attrition rates exceed 80% for both cohorts. Third, while the Young Lives data may lack national representativeness, their socioeconomic distribution closely mirrors that of nationally representative surveys, enhancing external validity. Lastly, the dataset includes objectively measured weight and height, enhancing reliability.^25^

Our study has several limitations. First, the small cohort sample size led to elevated SEs in some transition probabilities and uncertainty in the logistic regression model, especially for less frequent transitions. This prevented investigation of factors associated with transitions between different malnourished states (e.g. from stunting to overweight) in the four-state model. Instead, we fitted separate two-state models for each malnourished state. Second, we cannot differentiate between ageing effects and cohort effects in the unadjusted four-state model. Some transitions may be influenced by ageing and maturation, while others may be linked to changed food environments over the study period. Third, due to model requirements, only participants present in all rounds were included, and listwise deletion was used for missing data, potentially introducing bias. Supplementary Table S2, available as Supplementary data at *IJE* online, shows that the main differences between included and excluded participants are related to place of residence in India and Vietnam, with urban participants more likely to be lost to follow-up or have missing data. Fourth, in the adjusted two-state models, we did not adjust for some important factors such as maternal education, while some residual confounding may persist due to the observational nature of the data. Fifth, the pubertal timing in our sample may differ from the WHO growth reference, potentially leading to misclassification of stunting and overweight among early or late maturers.^47^ However, due to data constraints, we were unable to assess this impact. Finally, while the model incorporates time-varying confounders in transition probabilities at each assessment point, it offers an overall estimate across all rounds, limiting the ability to capture potential variations in associations over time.

In conclusion, childhood and adolescence emerge as critical periods for addressing stunting and overweight, thereby averting the development of CSO later in life. As nutrition transition advances, inequalities in the DBM appear to widen, with poorer groups facing a higher risk of persistent stunting and progressing transition rates into overweight. Our findings emphasise the necessity for age- and context-specific interventions to effectively address all forms of malnutrition.

## Ethics approval

For this secondary analysis, specific ethical approval was not required, as the data are de-identified and publicly accessible, ensuring that participants' privacy and confidentiality are maintained.

## Supplementary Material

dyae151_Supplementary_Data

## Data Availability

All data relevant to the study are included in the article or uploaded as supplementary information.
