# Supplementary material for: Longitudinal transitions of the double burden of overweight and stunting from childhood to early adulthood in India, Peru, and Vietnam
Source: Int J Epidemiol. 2024 Nov 14;53(6):dyae151. doi: 10.1093/ije/dyae151 (PMC11565240; doi:10.1093/ije/dyae151)
Supplement: dyae151_Supplementary_Data [file dyae151_supplementary_data.docx]

**Supplementary Materials**

[**Table S1.** Sample size in total and across models 2](#_Toc180588254)

[**Table S2.** Baseline characteristics of participants included in unadjusted four-state model, those excluded due to loss to follow-up, and those excluded due to missing anthropometric data. 3](#_Toc180588255)

[**Table S3.** Transition probabilities and standard errors between normal, stunting, overweight and concurrent stunting and overweight (CSO) over time for the Younger Cohort (YC) and Older Cohort (OC) from adjusted four-state model. 6](#_Toc180588256)

[**Table S4.** Akaike Information Criterion (AIC) for various model configurations of the two-state models, including models with univariate covariates, stepwise addition of covariates, and the final model including all covariates. 8](#_Toc180588257)

[**Table S5.** Odds ratios from univariable two-state models showing unadjusted association between covariates and likelihood of transitioning into and out of each nutritional state. 10](#_Toc180588258)

[**Table S6.** Odds ratios from final adjusted two-state models showing the associations between covariates and the likelihood of transitioning into and out of stunting, overweight and concurrent stunting and overweight (CSO). 12](#_Toc180588259)

[**Figure S1:** Marginal distribution probabilities of normal, stunted, overweight and concurrently stunted and overweight (CSO) for female and male children aged 1 to 15 (younger cohort (YC)). 14](#_Toc180588260)

[**Figure S2:** Transition probabilities between normal, stunted, overweight, concurrent stunting and overweight (CSO) state over time for females and males of the Younger Cohort (YC). Initial states are indicated in the columns and end states are indicated by colours. 15](#_Toc180588261)

[**Figure S3:** Odds Ratios and 95% CI showing the relative difference for transitions into and out of stunting (A), overweight (B), and concurrent stunting and overweight (CSO, C) by sex, household size, residence, wealth index quartile, and maternal education from adjusted two-state models. 16](#_Toc180588262)

# **Table S1.** Sample size in total and across models

|  |  |  | Unadjusted four-state model | Adjusted two-state models | Adjusted two-state models |
| --- | --- | --- | --- | --- | --- |
|  | Total (100%) | Present in all five rounds | Complete anthropometric data | Complete covariates | Complete maternal education data |
| India YC | 2011 | 1891 (94%) | 1829 (91%) | 1787 (89%) | 1784 (88.7%) |
| India OC | 1008 | 917 (91%) | 877 (87%) | 855 (85%) | 835 (83%) |
| Peru YC | 2052 | 1807 (88%) | 1750 (85%) | 1725 (84%) | 1630 (79%) |
| Peru OC | 714 | 580 (81%) | 540 (76%) | 531 (74%) | 471 (66%) |
| Vietnam YC | 2000 | 1891 (94.6%) | 1834 (92%) | 1766 (88%) | 1740 (87%) |
| Vietnam OC | 1000 | 830 (83%) | 808 (81%) | 743 (74%) | 717 (71%) |
| Total YC | 6063 | 5589 (92%) | 5413 (89%) | 5278 (87%) | 5154 (85%) |
| Total OC | 2722 | 2327 (86%) | 2225 (82%) | 2129 (78%) | 2023 (74%) |

# **Table S2.** Baseline characteristics of participants included in unadjusted four-state model, those excluded due to loss to follow-up, and those excluded due to missing anthropometric data. *P* value is from chi-squared test for the difference between included and total excluded.

|  |  | Included N (%) | Total excluded N (%) | | *P* |
| --- | --- | --- | --- | --- | --- |
|  |  |  | Loss to follow-up | Missing anthropometric data |  |
| India YC | | | | | |
| Residence | Urban | 444 (87.40%) | 57 (11.22%) | 7 (1.38%) | <0.001 |
|  | Rural | 1385 (92.15%) | 63 (4.19%) | 55 (3.66%) |  |
|  | Missing | 0 (0%) | 0 (0%) | 0 (0%) |  |
| Wealth | Q1 | 464 (90.80%) | 22 (4.31%) | 25 (4.89%) | 0.908 |
|  | Q2 | 459 (91.07%) | 26 (5.16%) | 19 (3.77%) |  |
|  | Q3 | 461 (91.47%) | 34 (6.75%) | 9 (1.79%) |  |
|  | Q4 | 440 (90.35%) | 38 (7.80%) | 9 (1.85%) |  |
|  | Missing | 5 (100.00%) | 0 (0%) | 0 (0%) |  |
| Sex | Female | 987 (91.30%) | 63 (5.83%) | 31 (2.87%) | 0.971 |
|  | Male | 842 (90.54%) | 57 (6.13%) | 31 (3.33%) |  |
|  | Missing | 0 (0%) | 0 (0%) | 0 (0%) |  |
| Household size | ≤5 years | 1116 (90.81%) | 78 (6.35%) | 35 (2.85%) | 0.620 |
|  | >5 years | 713 (91.18%) | 42 (5.37%) | 27 (3.45%) |  |
|  | Missing | 0 (0%) | 0 (0%) | 0 (0%) |  |
| India OC | | | | | |
| Residence | Urban | 200 (79.68%) | 39 (15.54%) | 12 (4.78%) | <0.001 |
|  | Rural | 677 (89.43%) | 52 (6.87%) | 28 (3.70%) |  |
|  | Missing | 0 (0%) | 0 (0%) | 0 (0%) |  |
| Wealth | Q1 | 228 (89.41%) | 14 (5.49%) | 13 (5.10%) | 0.019 |
|  | Q2 | 220 (97.30%) | 22 (8.73%) | 10 (3.97%) |  |
|  | Q3 | 220 (88.35%) | 21 (8.43%) | 8 (3.21%) |  |
|  | Q4 | 209 (82.94%) | 34 (13.49%) | 9 (3.57%) |  |
|  | Missing | 0 (0%) | 0 (0%) | 0 (0%) |  |
| Sex | Male | 436 (88.80%) | 45 (9.16%) | 10 (2.04%) | 0.795 |
|  | Female | 441 (85.30%) | 46 (8.90%) | 30 (5.80%) |  |
|  | Missing | 0 (0%) | 0 (0%) | 0 (0%) |  |
| Household size | ≤5 years | 542 (86.72%) | 60 (9.60%) | 23 (3.68%) | 0.373 |
|  | >5 years | 335 (87.47%) | 31 (8.09%) | 17 (4.44%) |  |
|  | Missing | 0 (0%) | 0 (0%) | 0 (0%) |  |
| Peru YC | | | | | |
| Residence | Urban | 1206 (85.78%) | 174 (12.38%) | 26 (1.85%) | 0.403 |
|  | Rural | 544 (84.21%) | 71 (10.99%) | 31 (4.80%) |  |
|  | Missing | 0 (0%) | 0 (0%) | 0 (0%) |  |
| Wealth | Q1 | 423 (82.14%) | 69 (13.40%) | 23 (4.47%) | 0.475 |
|  | Q2 | 439 (86.25%) | 55 (10.81%) | 15 (2.95%) |  |
|  | Q3 | 440 (85.94%) | 62 (12.11%) | 10 (1.95%) |  |
|  | Q4 | 444 (86.89%) | 58 (11.35%) | 9 (1.76%) |  |
|  | Missing | 4 (80.00%) | 1 (20.00%) | 0 (0.00%) |  |
| Sex | Male | 882 (85.88%) | 116 (11.30%) | 29 (2.82%) | 0344 |
|  | Female | 868 (84.68%) | 129 (12.59%) | 28 (2.73%) |  |
|  | Missing | 0 (0%) | 0 (0%) | 0 (0%) |  |
| Household size | ≤5 years | 963 (84.86%) | 134 (12.15%) | 33 (2.99%) | 0.572 |
|  | >5 years | 814 (85.77%) | 111 (11.70%) | 24 (2.53%) |  |
|  | Missing | 0 (0%) | 0 (0%) | 0 (0%) |  |
| Peru OC | | | | | |
| Residence | Urban | 411 (77.55%) | 87 (16.42%) | 32 (6.04%) | 0.013 |
|  | Rural | 129 (70.11%) | 47 (25.54%) | 8 (4.35%) |  |
|  | Missing | 0 (0%) | 0 (0%) | 0 (0%) |  |
| Wealth | Q1 | 124 (69.27%) | 43 (24.02%) | 0 (0.00%) | 0.147 |
|  | Q2 | 128 (73.14%) | 37 (21.14%) | 2 (1.14%) |  |
|  | Q3 | 142 (79.33%) | 27 (15.08%) | 1 (0.56%) |  |
|  | Q4 | 141 (80.57%) | 26 (14.86%) | 1 (0.57%) |  |
|  | Missing | 5 (83.33%) | 1 (16.67%) |  |  |
| Sex | Male | 208 (72.54%) | 79 (20.47%) | 27 (6.99%) | 0.305 |
|  | Female | 260 (79.27%) | 55 (16.77%) | 13 (3.96%) |  |
|  | Missing | 0 (0%) | 0 (0%) | 0 (0%) |  |
| Household size | ≤5 years | 303 (79.53%) | 63 (16.54%) | 15 (3.94%) | 0.067 |
|  | >5 years | 237 (71.17%) | 71 (21.32%) | 25 (7.51%) |  |
|  | Missing | 0 (0%) | 0 (0%) | 0 (0%) |  |
| Vietnam YC | | | | | |
| Residence | Urban | 333 (83.25%) | 54 (13.50%) | 13 (3.25%) | <0.001 |
|  | Rural | 1501 (93.81%) | 55 (3.44%) | 44 (2.75%) |  |
|  | Missing | 0 (0%) | 0 (0%) | 0 (0%) |  |
| Wealth | Q1 | 456 (91.02%) | 30 (5.99%) | 15 (2.99%) | <0.001 |
|  | Q2 | 479 (95.99%) | 9 (1.80%) | 11 (2.20%) |  |
|  | Q3 | 595 (94.11%) | 16 (3.04%) | 15 (2.85%) |  |
|  | Q4 | 404 (85.41%) | 53 (11.21%) | 16 (3.38%) |  |
|  | Missing | 0 (0%) | 1 (100%) | 0 (0%) |  |
| Sex | Male | 938 (91.33%) | 58 (5.65%) | 31 (3.02%) | 0.511 |
|  | Female | 896 (92.09%) | 51 (5.24%) | 26 (2.67%) |  |
|  | Missing | 0 (0%) | 0 (0%) | 0 (0%) |  |
| Household size | ≤5 years | 1301 (91.62%) | 80 (5.63%) | 39 (2.75%) | 0.730 |
|  | >5 years | 533 (91.90%) | 29 (5.00%) | 18 (3.10%) |  |
|  | Missing | 0 (0%) | 0 (0%) | 0 (0%) |  |
| Vietnam OC | | | | | |
| Residence | Urban | 148 (74.00%) | 50 (25.00%) | 2 (1.00%) | 0.001 |
|  | Rural | 660 (82.50%) | 120 (15.00%) | 20 (2.50%) |  |
|  | Missing | 0 (0%) | 0 (0%) | 0 (0%) |  |
| Wealth | Q1 | 203 (80.88%) | 44 (17.53%) | 4 (1.59%) | 0.094 |
|  | Q2 | 213 (85.54%) | 31 (12.45%) | 5 (2.01%) |  |
|  | Q3 | 209 (80.08% | 43 (16.48%) | 9 (3.45%) |  |
|  | Q4 | 182 (76.47%) | 52 (21.85%) | 4 (1.68%) |  |
|  | Missing | 1 (100.00%) | 0 (0%) | 0 (0%) |  |
| Sex | Female | 381 (76.05%) | 110 (21.96%) | 10 (2.00%) | <0.001 |
|  | Male | 427 (85.57%) | 60 (12.02%) | 12 (2.40%) |  |
|  | Missing | 0 (0%) | 0 (0%) | 0 (0%) |  |
| Household size | ≤5 years | 596 (81.76%) | 120 (16.46%) | 13 (1.78%) | 0.457 |
|  | >5 years | 212 (78.23) | 50 (18.45%) | 9 (3.32%) |  |
|  | Missing | 0 (0%) | 0 (0%) | 0 (0%) |  |

*Abbreviations:* YC, Younger Cohort; OC, Older Cohort; CSO, concurrent stunting and overweight.

*Note:* Wealth levels indicate quartiles of a wealth index (Q1 = poorest).

# **Table S3.** Transition probabilities and standard errors between normal, stunting, overweight and concurrent stunting and overweight (CSO) over time for the Younger Cohort (YC) and Older Cohort (OC) from adjusted four-state model.

| Initial state | End state | 1-5y | 5-8y | 8-12y | 12-15y | 8-12y | 12-15y | 15-19y | | 19-22y | |  |
| --- | --- | --- | --- | --- | --- | --- | --- | --- | --- | --- | --- | --- |
| India | | **YC** | | | | **OC** | | | | | |  |
| Normal | Normal | 0.76 (0.01) | 0.92 (0.01) | 0.85 (0.01) | 0.84 (0.01) | 0.84 (0.02) | 0.81 (0.02) | 0.84 (0.02) | | 0.84 (0.02) | |  |
| Normal | Stunted | 0.23 (0.01) | 0.06 (0.01) | 0.09 (0.01) | 0.12 (0.01) | 0.13 (0.01) | 0.17 (0.02) | 0.14 (0.01) | | 0.05 (0.01) | |  |
| Normal | Overweight | 0.01 (0) | 0.02 (0) | 0.05 (0.01) | 0.03 (0.01) | 0.03 (0.01) | 0.01 (0) | 0.02 (0.01) | | 0.10 (0.01) | |  |
| Normal | CSO | 0 (0) | 0 (0) | 0.01 (0) | 0.01 (0) | 0 (0) | 0 (0) | 0.01 (0) | | 0.01 (0) | |  |
| Stunted | Normal | 0.34 (0.02) | 0.29 (0.02) | 0.25 (0.02) | 0.35 (0.02) | 0.25 (0.03) | 0.28 (0.03) | 0.37 (0.03) | | 0.21 (0.03) | |  |
| Stunted | Stunted | 0.66 (0.02) | 0.7 (0.02) | 0.75 (0.02) | 0.63 (0.02) | 0.74 (0.03) | 0.7 (0.03) | 0.6 (0.03) | | 0.72 (0.03) | |  |
| Stunted | Overweight | 0 (0) | 0 (0) | 0 (0) | 0 (0) | 0.01 (0.01) | 0.01 (0.01) | 0 (0) | | 0.02 (0.01) | |  |
| Stunted | CSO | 0 (0) | 0 (0) | 0 (0) | 0.01 (0) | 0 (0) | 0 (0) | 0.03 (0.01) | | 0.04 (0.01) | |  |
| Overweight | Normal | 0.75 (0.22) | 0.43 (0.13) | 0.23 (0.08) | 0.31 (0.05) | 0.22 (0.14) | 0.34 (0.09) | 0.32 (0.09) | | 0.13 (0.06) | |  |
| Overweight | Stunted | 0 (0) | 0.14 (0.09) | 0.03 (0.03) | 0.03 (0.02) | 0 (0) | 0.07 (0.05) | 0 (0) | | 0.03 (0.03) | |  |
| Overweight | Overweight | 0.25 (0.22) | 0.43 (0.13) | 0.71 (0.08) | 0.56 (0.05) | 0.78 (0.14) | 0.55 (0.09) | 0.64 (0.1) | | 0.77 (0.08) | |  |
| Overweight | CSO | 0 (0) | 0 (0) | 0.03 (0.03) | 0.10 (0.03) | 0 (0) | 0.03 (0.03) | 0.04 (0.04) | | 0.07 (0.05) | |  |
| CSO | Normal | 0.50 (0.25) | 0.40 (0.22) | 0.33 (0.19) | 0.36 (0.15) | 0.50 (0.35) | 0 (0) | 0.20 (0.18) | | 0.14 (0.09) | |  |
| CSO | Stunted | 0.50 (0.25) | 0.20 (0.18) | 0.33 (0.19) | 0.09 (0.09) | 0.50 (0.35) | 0 (0) | 0.20 (0.18) | | 0.14 (0.09) | |  |
| CSO | Overweight | 0 (0) | 0.20 (0.18) | 0 (0) | 0.27 (0.13) | 0 (0) | 0 (0) | 0.20 (0.18) | | 0.14 (0.09) | |  |
| CSO | CSO | 0 (0) | 0.20 (0.18) | 0.33 (0.19) | 0.27 (0.13) | 0 (0) | 1 (0) | 0.40 (0.22) | | 0.57 (0.13) | |  |
| Peru | | **YC** | | | | **OC** | | | | | |  |
| Normal | Normal | 0.6 (0.01) | 0.76 (0.01) | 0.75 (0.01) | 0.92 (0.01) | 0.75 (0.03) | 0.83 (0.02) | 0.71 (0.03) | | 0.73 (0.03) | |  |
| Normal | Stunted | 0.18 (0.01) | 0.03 (0.01) | 0.07 (0.01) | 0.07 (0.01) | 0.14 (0.02) | 0.07 (0.01) | 0.12 (0.02) | | 0.05 (0.01) | |  |
| Normal | Overweight | 0.18 (0.01) | 0.20  (0.01) | 0.17 (0.01) | 0.02 (0) | 0.11 (0.02) | 0.09 (0.02) | 0.14 (0.02) | | 0.20 (0.02) | |  |
| Normal | CSO | 0.05 (0.01) | 0.01 (0) | 0.01 (0) | 0 (0) | 0 (0) | 0.01 (0.01) | 0.03 (0.01) | | 0.01 (0.01) | |  |
| Stunted | Normal | 0.25 (0.02) | 0.40 (0.02) | 0.22 (0.03) | 0.40 (0.03) | 0.22 (0.04) | 0.38 (0.04) | 0.21 (0.04) | | 0.10 (0.03) | |  |
| Stunted | Stunted | 0.57 (0.03) | 0.50 (0.02) | 0.70 (0.03) | 0.59 (0.03) | 0.71 (0.04) | 0.56 (0.04) | 0.60 (0.05) | | 0.66 (0.05) | |  |
| Stunted | Overweight | 0.04 (0.01) | 0.04 (0.01) | 0.04 (0.01) | 0 (0) | 0.01 (0.01) | 0.01 (0.01) | 0.06 (0.02) | | 0.04 (0.02) | |  |
| Stunted | CSO | 0.14 (0.02) | 0.06 (0.01) | 0.03 (0.01) | 0 (0) | 0.06 (0.02) | 0.05 (0.02) | 0.14 (0.03) | | 0.20 (0.04) | |  |
| Overweight | Normal | 0.45 (0.04) | 0.28 (0.03) | 0.19 (0.02) | 0.59 (0.02) | 0.27 (0.04) | 0.31 (0.05) | 0.22 (0.04) | | 0.21 (0.04) | |  |
| Overweight | Stunted | 0.07 (0.02) | 0 (0) | 0.02 (0.01) | 0.02 (0.01) | 0.05 (0.02) | 0.02 (0.01) | 0.03 (0.02) | | 0.02 (0.01) | |  |
| Overweight | Overweight | 0.42 (0.04) | 0.71 (0.03) | 0.79 (0.02) | 0.36 (0.02) | 0.64 (0.05) | 0.61 (0.05) | 0.60 (0.05) | | 0.73 (0.04) | |  |
| Overweight | CSO | 0.07 (0.02) | 0.01 (0) | 0.01 (0) | 0.03 (0.01) | 0.04 (0.02) | 0.07 (0.02) | 0.15 (0.04) | | 0.04 (0.02) | |  |
| CSO | Normal | 0.35 (0.08) | 0.37 (0.04) | 0.33 (0.07) | 0.46 (0.1) | 0.18 (0.07) | 0.13 (0.09) | 0 (0) | | 0.06 (0.03) | |  |
| CSO | Stunted | 0.38 (0.08) | 0.30 (0.04) | 0.39 (0.07) | 0.38 (0.1) | 0.48 (0.09) | 0.40 (0.13) | 0.29 (0.1) | | 0.14 (0.05) | |  |
| CSO | Overweight | 0.15 (0.06) | 0.22 (0.04) | 0.16 (0.05) | 0.04 (0.04) | 0.18 (0.07) | 0.27 (0.11) | 0.05 (0.05) | | 0.24 (0.06) | |  |
| CSO | CSO | 0.12 (0.06) | 0.11 (0.03) | 0.12 (0.05) | 0.13 (0.07) | 0.15 (0.06) | 0.20 (0.1) | 0.67 (0.1) | | 0.57 (0.07) | |  |
| Vietnam | | **YC** |  |  |  | **OC** | | | | | |  |
| Normal | Normal | 0.78 (0.01) | 0.89 (0.01) | 0.85 (0.01) | 0.92 (0.01) | 0.83 (0.02) | 0.91 (0.01) | 0.88 (0.01) | | 0.91 (0.01) | |  |
| Normal | Stunted | 0.15 (0.01) | 0.04 (0.01) | 0.09 (0.01) | 0.05 (0.01) | 0.12 (0.01) | 0.08 (0.01) | 0.11 (0.01) | | 0.05 (0.01) | |  |
| Normal | Overweight | 0.07 (0.01) | 0.06 (0.01) | 0.06 (0.01) | 0.03 (0) | 0.05 (0.01) | 0.02 (0.01) | 0.01 (0.01) | | 0.04 (0.01) | |  |
| Normal | CSO | 0 (0) | 0.01 (0) | 0 (0) | 0 (0) | 0 (0) | 0 (0) | 0 (0) | | 0 (0) | |  |
| Stunted | Normal | 0.23 (0.03) | 0.34 (0.02) | 0.33 (0.03) | 0.56 (0.03) | 0.20 (0.03) | 0.42 (0.03) | 0.39 (0.04) | | 0.44 (0.04) | |  |
| Stunted | Stunted | 0.74 (0.03) | 0.65 (0.02) | 0.65 (0.03) | 0.43 (0.03) | 0.80 (0.03) | 0.57 (0.03) | 0.58 (0.04) | | 0.49 (0.04) | |  |
| Stunted | Overweight | 0.01 (0.01) | 0 (0) | 0.01 (0.01) | 0.01 (0.01) | 0 (0) | 0 (0) | 0.02 (0.01) | | 0.01 (0.01) | |  |
| Stunted | CSO | 0.02 (0.01) | 0.01 (0) | 0.01 (0) | 0 (0) | 0 (0) | 0 (0) | 0.01 (0.01) | | 0.06 (0.02) | |  |
| Overweight | Normal | 0.44 (0.12) | 0.23 (0.04) | 0.29 (0.04) | 0.42 (0.04) | 0.38 (0.12) | 0.46 (0.08) | 0.48 (0.1) | | 0.24 (0.09) | |  |
| Overweight | Stunted | 0.06 (0.06) | 0.02 (0.01) | 0.01 (0.01) | 0.01 (0.01) | 0 (0) | 0.09 (0.05) | | 0.04 (0.04) | | 0 (0) | |
| Overweight | Overweight | 0.44 (0.12) | 0.74 (0.04) | 0.69 (0.04) | 0.55 (0.04) | 0.63 (0.12) | 0.40 (0.08) | 0.43 (0.1) | | 0.76 (0.09) | |  |
| Overweight | CSO | 0.06 (0.06) | 0.01 (0.01) | 0.01 (0.01) | 0.02 (0.01) | 0 (0) | 0.06 (0.04) | 0.04 (0.04) | | 0 (0) | |  |
| CSO | Normal | 0.25 (0.22) | 0.18 (0.12) | 0.41 (0.12) | 0.40 (0.15) | 0 (0) | 0 (0) | 0.20 (0.18) | | 0.50 (0.25) | |  |
| CSO | Stunted | 0.75 (0.22) | 0.73 (0.13) | 0.18 (0.09) | 0.10 (0.09) | 0.67 (0.27) | 0 (0) | 0.40 (0.22) | | 0 (0) | |  |
| CSO | Overweight | 0 (0) | 0 (0) | 0.41 (0.12) | 0.10 (0.09) | 0.33 (0.27) | 0 (0) | 0 (0) | | 0.25 (0.22) | |  |
| CSO | CSO | 0 (0) | 0.09 (0.09) | 0 (0) | 0.40 (0.15) | 0 (0) | 1 (0) | 0.40 (0.22) | | 0.25 (0.22) | |  |

*Abbreviations:* YC, Younger Cohort; OC, Older Cohort; CSO, concurrent stunting and overweight.

# **Table S4.** Akaike Information Criterion (AIC) for various model configurations of the two-state models, including models with univariate covariates, stepwise addition of covariates, and the final model including all covariates.

| **Peru YC** | **Stunting** | **Overweight** | **CSO** |
| --- | --- | --- | --- |
| Unadjusted | 7153.11933 | 7825.40072 | 2185.67239 |
| Wealth (quartiles) | 6800.05999 | 7612.20593 | 2182.08931 |
| Sex (male/female) | 7134.17805 | 7807.72988 | 2184.9475 |
| Residence (urban/rural) | 6883.17421 | 7696.30794 | **2180.62235** |
| Household size (≤5/>5) | 7113.68413 | 7806.80202 | 2188.19933 |
| Wealth & residence | 6759.96651 | 7602.47355 | 2186.33635 |
| Wealth, residence & sex | 6742.22788 | 7584.73127 | 2185.55399 |
| Wealth, residence, sex & household size (final model) | **6728.13287** | **7581.11007** | 2187.67964 |
| **Peru OC** | **Stunting** | **Overweight** | **CSO** |
| Unadjusted | 2344.7024 | 2585.61337 | 1175.64128 |
| Wealth (quartiles) | 2300.31716 | 2583.84637 | 1188.99324 |
| Sex (male/female) | 2349.03224 | 2583.20332 | 1174.18454 |
| Residence (urban/rural) | 2320.70174 | 2569.57334 | 1176.69763 |
| Household size (≤5/>5) | 2326.687 | 2570.59354 | **1171.74475** |
| Wealth & residence | 2304.33212 | 2581.61794 | 1191.93481 |
| Wealth, residence & sex | 2307.31111 | 2578.58264 | 1190.11989 |
| Wealth, residence, sex & household size (final model) | **2299.15148** | **2570.11432** | 1188.40961 |
| **India YC** | **Stunting** | **Overweight** | **CSO** |
| Unadjusted | 8546.38011 | 1981.05455 | **595.565652** |
| Wealth (quartiles) | 8401.85845 | 1930.02701 | 600.408189 |
| Sex (male/female) | 8542.34073 | 1985.75958 | 596.027177 |
| Residence (urban/rural) | 8468.69268 | **1901.22486** | 591.516415 |
| Household size (≤5/>5) | 8548.84731 | 1978.15552 | 598.11403 |
| Wealth & residence | 8398.68875 | 1902.57015 | 597.949585 |
| Wealth, residence & sex | **8392.1658** | 1907.27136 | 601.505959 |
| Wealth, residence, sex & household size (final model) | 8395.97722 | 1906.56156 | 604.841102 |
| **India OC** | **Stunting** | **Overweight** | **CSO** |
| Unadjusted | 4141.20931 | 1399.11544 | **445.495831** |
| Wealth (quartiles) | 4117.94168 | 1352.26671 | 457.242162 |
| Sex (male/female) | 4129.86269 | 1404.47312 | 447.320721 |
| Residence (urban/rural) | 4115.34679 | 1357.83979 | 447.579526 |
| Household size (≤5/>5) | 4138.17393 | 1396.74564 | 444.859639 |
| Wealth & residence | 4115.24421 | 1347.88005 | 459.52775 |
| Wealth, residence & sex | 4102.13387 | 1352.90145 | 461.79384 |
| Wealth, residence, sex & household size (final model) | **4101.02114** | **1352.0076** | 459.820396 |
| **Vietnam YC** | **Stunting** | **Overweight** | **CSO** |
| Unadjusted | 6553.50015 | 3514.07555 | **627.597605** |
| Wealth (quartiles) | 6514.66652 | 3390.62456 | 630.577385 |
| Sex (male/female) | 6374.54677 | 3492.45814 | 632.544586 |
| Residence (urban/rural) | 6552.24971 | 3361.8978 | 629.846382 |
| Household size (≤5/>5) | 6475.35096 | 3517.08307 | 630.250574 |
| Wealth & residence | 6368.74899 | 3340.82749 | 634.267223 |
| Wealth, residence & sex | 6364.35246 | 3317.81698 | 639.063485 |
| Wealth, residence, sex & household size (final model) | **6325.80765** | **3321.58487** | 641.90763 |
| **Vietnam OC** | **Stunting** | **Overweight** | **CSO** |
| Unadjusted | 3220.33922 | 1070.06677 | 293.00478 |
| Wealth (quartiles) | 3209.51148 | 1046.1494 | 293.083754 |
| Sex (male/female) | 3193.72506 | 1065.03905 | 298.215669 |
| Residence (urban/rural) | 3215.42472 | 1038.14323 | **291.064581** |
| Household size (≤5/>5) | 3202.97111 | 1075.04106 | 291.431581 |
| Wealth & residence | 3212.43925 | 1034.3398 | 293.480541 |
| Wealth, residence & sex | 3187.25701 | **1031.71141** | 297.613766 |
| Wealth, residence, sex & household size (final model) | **3167.61914** | 1037.3287 | 296.732562 |

*Abbreviations:* YC, Younger Cohort; OC, Older Cohort; CSO, concurrent stunting and overweight.

# **Table S5.** Odds ratios from univariable two-state models showing unadjusted association between covariates and likelihood of transitioning into and out of each nutritional state.

|  |  | Stunting |  | Overweight | | CSO |  |
| --- | --- | --- | --- | --- | --- | --- | --- |
| Country and cohort | Variable | Transitions into (OR 95% CI) | Transitions out of (OR 95% CI) | Transitions into (OR 95% CI) | Transitions out of (OR 95% CI) | Transitions into (OR 95% CI) | Transitions out of (OR 95% CI) |
| Vietnam YC | Wealth Q1 | 1 (ref) | 1 (ref) | 1 (ref) | 1 (ref) | 1 (ref) | 1 (ref) |
|  | Wealth Q2 | 0.71 (0.57; 0.88) | 1.13 (0.88; 1.45) | 2.11 (1.14; 3.93) | 0.63 (0.15; 2.66) | 1.01 (0.35; 2.89) | NA |
|  | Wealth Q3 | 0.65 (0.52; 0.8) | 1.82 (1.42; 2.34) | 3.29 (1.84; 5.91) | 0.4 (0.11; 1.44) | 2.14 (0.87; 5.27) | NA |
|  | Wealth Q4 | 0.37 (0.29; 0.48) | 2.17 (1.62; 2.91) | 6.22 (3.57; 10.83) | 0.34 (0.1; 1.14) | 1.77 (0.69; 4.5) | NA |
|  | Rural residence | 1.78 (1.46; 2.16) | 0.57 (0.45; 0.72) | 0.73 (0.54; 0.98) | 1.77 (0.94; 3.32) | 0.39 (0.21; 0.71) | NA |
|  | Female | 1.07 (0.91; 1.26) | 1.22 (1.01; 1.47) | 1.02 (0.75; 1.37) | 0.72 (0.38; 1.35) | 0.83 (0.45; 1.55) | NA |
|  | Household size >5 | 1.17 (0.99; 1.38) | 1.01 (0.84; 1.22) | 0.73 (0.54; 0.98) | 1.77 (0.94; 3.32) | 0.69 (0.37; 1.27) | NA |
| Vietnam OC | Wealth Q1 | 1 (ref) | 1 (ref) | 1 (ref) | 1 (ref) | 1 (ref) | 1 (ref) |
|  | Wealth Q2 | 0.87 (0.63; 1.21) | 1.07 (0.76; 1.51) | 2.21 (1.07; 4.57) | 0.84 (0.18; 3.88) | 2.21 (1.07; 4.57) | NA |
|  | Wealth Q3 | 0.53 (0.37; 0.76) | 1.35 (0.94; 1.92) | 3.82 (1.94; 7.51) | 0.38 (0.09; 1.64) | 3.82 (1.94; 7.51) | NA |
|  | Wealth Q4 | 0.48 (0.33; 0.68) | 1.39 (0.95; 2.03) | 6.8 (3.56; 13.01) | 0.46 (0.12; 1.71) | 6.8 (3.56; 13.01) | NA |
|  | Rural residence | 1.56 (1.16; 2.08) | 0.63 (0.46; 0.86) | 0.32 (0.23; 0.45) | 1.66 (0.74; 3.71) | 0.32 (0.23; 0.45) | NA |
|  | Female | 1.37 (1.07; 1.76) | 0.64 (0.5; 0.84) | 0.89 (0.63; 1.26) | 0.87 (0.39; 1.92) | 0.89 (0.63; 1.26) | NA |
|  | Household size >5 | 1.22 (0.95; 1.57) | 0.72 (0.55; 0.93) | 0.6 (0.42; 0.85) | 0.93 (0.42; 2.06) | 0.6 (0.42; 0.85) | NA |
| Peru YC | Wealth Q1 | 1 (ref) | 1 (ref) | 1 (ref) | 1 (ref) | 1 (ref) | 1 (ref) |
|  | Wealth Q2 | 0.55 (0.44; 0.7) | 1.15 (0.9; 1.47) | 1.61 (1.27; 2.05) | 0.58 (0.41; 0.83) | 0.98 (0.68; 1.41) | NA |
|  | Wealth Q3 | 0.26 (0.2; 0.34) | 2.43 (1.78; 3.33) | 2.15 (1.7; 2.72) | 0.35 (0.25; 0.49) | 0.48 (0.31; 0.75) | NA |
|  | Wealth Q4 | 0.17 (0.12; 0.22) | 2.92 (2; 4.25) | 3.26 (2.59; 4.1) | 0.27 (0.19; 0.37) | 0.57 (0.37; 0.86) | NA |
|  | Rural residence | **3.64 (3.01; 4.41)** | **0.56 (0.45; 0.69)** | 0.51 (0.42; 0.61) | 3.08 (2.28; 4.14) | 1.68 (1.24; 2.27) | NA |
|  | Female | 1.38 (1.14; 1.66) | 1.1 (0.89; 1.35) | 0.73 (0.63; 0.85) | 0.76 (0.62; 0.93) | 0.77 (0.57; 1.03) | NA |
|  | Household size >5 | 1.56 (1.28; 1.9) | 0.71 (0.56; 0.89) | 0.75 (0.64; 0.88) | 1.41 (1.16; 1.72) | 1.13 (0.83; 1.54) | NA |
| Peru OC | Wealth Q1 | 1 (ref) | 1 (ref) | 1 (ref) | 1 (ref) | 1 (ref) | 1 (ref) |
|  | Wealth Q2 | 0.74 (0.48; 1.12) | 1.23 (0.78; 1.95) | 1.26 (0.85; 1.88) | 0.75 (0.43; 1.32) | 1.26 (0.85; 1.88) | NA |
|  | Wealth Q3 | 0.4 (0.25; 0.63) | 1.26 (0.75; 2.12) | 1.42 (0.96; 2.1) | 0.56 (0.32; 0.99) | 1.42 (0.96; 2.1) | NA |
|  | Wealth Q4 | 0.4 (0.25; 0.64) | 1.2 (0.67; 2.14) | 1.31 (0.88; 1.95) | 0.58 (0.33; 1.01) | 1.31 (0.88; 1.95) | NA |
|  | Rural residence | **1.84 (1.24; 2.73)** | **0.82 (0.54; 1.24)** | 0.64 (0.44; 0.93) | 2.12 (1.24; 3.62) | 0.64 (0.44; 0.93) | NA |
|  | Female | 0.93 (0.68; 1.28) | 1.12 (0.77; 1.62) | 1.29 (0.97; 1.7) | 0.74 (0.51; 1.08) | 1.29 (0.97; 1.7) | NA |
|  | Household size >5 | 1.46 (1.05; 2.03) | 1.05 (0.72; 1.55) | 0.68 (0.52; 0.9) | 1.39 (0.96; 2.02) | 0.68 (0.52; 0.9) | NA |
| Vietnam YC | Wealth Q1 | 1 (ref) | 1 (ref) | 1 (ref) | 1 (ref) | 1 (ref) | 1 (ref) |
|  | Wealth Q2 | 0.44 (0.34; 0.57) | 1.32 (1; 1.73) | 0.75 (0.5; 1.12) | 0.58 (0.29; 1.15) | 0.29 (0.11; 0.79) | NA |
|  | Wealth Q3 | 0.5 (0.4; 0.64) | 2.06 (1.54; 2.77) | 1.44 (1.01; 2.04) | 0.55 (0.29; 1.04) | 0.81 (0.4; 1.64) | NA |
|  | Wealth Q4 | 0.27 (0.2; 0.36) | 2.34 (1.59; 3.44) | 3.6 (2.63; 4.94) | 0.41 (0.23; 0.71) | 0.5 (0.21; 1.15) | NA |
|  | Rural residence | 2.42 (1.81; 3.23) | 0.49 (0.32; 0.73) | 0.23 (0.18; 0.29) | 1.78 (1.24; 2.55) | 1.28 (0.57; 2.88) | NA |
|  | Female | 0.96 (0.8; 1.16) | 1.09 (0.87; 1.36) | 0.59 (0.47; 0.74) | 1.46 (1.01; 2.13) | 0.73 (0.4; 1.34) | NA |
|  | Household size >5 | 1.83 (1.52; 2.21) | 0.79 (0.63; 0.98) | 0.95 (0.75; 1.19) | 1.06 (0.73; 1.54) | 1.46 (0.81; 2.64) | NA |
| Vietnam OC | Wealth Q1 | 1 (ref) | 1 (ref) | 1 (ref) | 1 (ref) | 1 (ref) | 1 (ref) |
|  | Wealth Q2 | 0.88 (0.59; 1.32) | 1.32 (0.9; 1.92) | 0.49 (0.22; 1.11) | 0.24 (0.05; 1.07) | 0.49 (0.22; 1.11) | NA |
|  | Wealth Q3 | 0.71 (0.47; 1.06) | 1.75 (1.17; 2.61) | 0.86 (0.44; 1.7) | 0.37 (0.11; 1.3) | 0.86 (0.44; 1.7) | NA |
|  | Wealth Q4 | 0.58 (0.37; 0.89) | 1.46 (0.93; 2.28) | 2.44 (1.38; 4.32) | 0.4 (0.14; 1.16) | 2.44 (1.38; 4.32) | NA |
|  | Rural residence | 1.61 (1.12; 2.31) | 0.84 (0.57; 1.22) | 0.28 (0.18; 0.44) | 0.83 (0.39; 1.76) | 0.28 (0.18; 0.44) | NA |
|  | Female | 1.22 (0.9; 1.64) | 2.06 (1.53; 2.78) | 0.72 (0.46; 1.13) | 1.66 (0.77; 3.57) | 0.72 (0.46; 1.13) | NA |
|  | Household size >5 | 1.65 (1.23; 2.22) | 0.87 (0.65; 1.17) | 1.21 (0.78; 1.89) | 1.25 (0.57; 2.78) | 1.21 (0.78; 1.89) | NA |

*Abbreviations:* YC, Younger Cohort; OC, Older Cohort; CSO, concurrent stunting and overweight.

*Note:* Wealth levels indicate quartiles of a wealth index (Q1 = poorest).

# **Table S6.** Odds ratios from final adjusted two-state models showing the associations between covariates and the likelihood of transitioning into and out of stunting, overweight and concurrent stunting and overweight (CSO).

|  |  | Stunting | | Overweight | | CSO | |
| --- | --- | --- | --- | --- | --- | --- | --- |
| Country and cohort | Variable | Transitions into  (OR 95% CI) | Transitions out of  (OR 95% CI) | Transitions into  (OR 95% CI) | Transitions out of  (OR 95% CI) | Transitions into  (OR 95% CI) | Transitions out of  (OR 95% CI) |
| India YC | Wealth Q1 | 1 (ref) | 1 (ref) | 1 (ref) | 1 (ref) | 1 (ref) | 1 (ref) |
|  | Wealth Q2 | 0.71 (0.57; 0.89) | 1.13 (0.88; 1.45) | 2.01 (1.08; 3.75) | 0.57 (0.13; 2.54) | 0.98 (0.34; 2.81) | NA |
|  | Wealth Q3 | 0.68 (0.54; 0.85) | 1.75 (1.35; 2.26) | 2.39 (1.31; 4.36) | 0.42 (0.11; 1.64) | 1.57 (0.61; 4.04) | NA |
|  | Wealth Q4 | 0.43 (0.32; 0.57) | 1.93 (1.39; 2.68) | 2.99 (1.61; 5.54) | 0.4 (0.11; 1.50) | 0.88 (0.30; 2.60) | NA |
|  | Rural residence | 1.23 (0.97; 1.56) | 0.75 (0.57; 0.99) | 0.35 (0.24; 0.51) | 1.25 (0.59; 2.63) | 0.38 (0.18; 0.80) | NA |
|  | Female | 1.06 (0.9; 1.25) | 1.28 (1.05; 1.54) | 1.06 (0.78; 1.43) | 0.70 (0.37; 1.35) | 0.86 (0.46; 1.60) | NA |
|  | Household size >5 | 1.03 (0.86; 1.23) | 1.02 (0.83; 1.25) | 0.88 (0.62; 1.25) | 1.92 (0.91; 4.09) | 0.71 (0.34; 1.50) | NA |
| India OC | Wealth Q1 | 1 (ref) | 1 (ref) | 1 (ref) | 1 (ref) | 1 (ref) | 1 (ref) |
|  | Wealth Q2 | 0.86 (0.62; 1.2) | 1 (0.7; 1.42) | 2.12 (1.03; 4.39) | 0.94 (0.20; 4.53) | 1.37 (0.47; 3.98) | NA |
|  | Wealth Q3 | 0.54 (0.38; 0.78) | 1.21 (0.83; 1.76) | 3.17 (1.59; 6.34) | 0.43 (0.1; 1.92) | 1.22 (0.41; 3.63) | NA |
|  | Wealth Q4 | 0.51 (0.34; 0.77) | 1.07 (0.69; 1.68) | 4.45 (2.19; 9.07) | 0.62 (0.14; 2.77) | 1.62 (0.52; 5.05) | NA |
|  | Rural residence | 1.13 (0.80; 1.61) | 0.65 (0.45; 0.94) | 0.54 (0.36; 0.82) | 1.51 (0.57; 4.03) | 0.73 (0.32; 1.71) | NA |
|  | Female | 1.41 (1.1; 1.81) | 0.63 (0.48; 0.82) | 0.87 (0.61; 1.23) | 0.82 (0.36; 1.86) | 1.43 (0.72; 2.85) | NA |
|  | Household size >5 | 1.07 (0.81; 1.42) | 0.84 (0.63; 1.13) | 0.75 (0.49; 1.16) | 1.17 (0.43; 3.24) | 0.37 (0.13; 1.07) | NA |
| Peru YC | Wealth Q1 | 1 (ref) | 1 (ref) | 1 (ref) | 1 (ref) | 1 (ref) | 1 (ref) |
|  | Wealth Q2 | 0.77 (0.6; 1.00) | 1.06 (0.82; 1.38) | 1.48 (1.15; 1.91) | 0.75 (0.51; 1.11) | 1.10 (0.74; 1.64) | 1.35 (0.45; 4.09) |
|  | Wealth Q3 | 0.44 (0.32; 0.60) | 2.00 (1.40; 2.86) | 1.89 (1.43; 2.50) | 0.51 (0.34; 0.75) | 0.59 (0.35; 1.00) | 1.27 (0.34; 4.69) |
|  | Wealth Q4 | 0.29 (0.20; 0.41) | 2.39 (1.57; 3.65) | 2.84 (2.14; 3.77) | 0.40 (0.27; 0.59) | 0.70 (0.42; 1.18) | 1.20 (0.29; 4.98) |
|  | Rural residence | 1.90 (1.49; 2.42) | 0.79 (0.61; 1.02) | 0.84 (0.66; 1.06) | 1.82 (1.27; 2.61) | 1.27 (0.86; 1.87) | 1.09 (0.38; 3.11) |
|  | Female | 1.32 (1.09; 1.60) | 1.09 (0.88; 1.34) | 0.73 (0.62; 0.85) | 0.75 (0.61; 0.92) | 0.77 (0.57; 1.03) | 0.46 (0.20; 1.07) |
|  | Household size >5 | 1.47 (1.21; 1.78) | 0.82 (0.66; 1.01) | 0.95 (0.80; 1.11) | 1.35 (1.09; 1.67) | 1.24 (0.92; 1.68) | 1.33 (0.58; 3.08) |
| Peru OC | Wealth Q1 | 1 (ref) | 1 (ref) | 1 (ref) | 1 (ref) | 1 (ref) | 1 (ref) |
|  | Wealth Q2 | 0.78 (0.49; 1.23) | 1.16 (0.71; 1.92) | 1.09 (0.71; 1.68) | 0.84 (0.46; 1.56) | 1.04 (0.56; 1.93) | 0.48 (0.16; 1.44) |
|  | Wealth Q3 | 0.43 (0.25; 0.72) | 1.15 (0.63; 2.1) | 1.15 (0.73; 1.80) | 0.67 (0.35; 1.27) | 0.77 (0.39; 1.51) | 0.92 (0.27; 3.15) |
|  | Wealth Q4 | 0.43 (0.26; 0.72) | 1.11 (0.58; 2.11) | 1.06 (0.67; 1.68) | 0.69 (0.36; 1.32) | 0.87 (0.45; 1.70) | 0.57 (0.16; 2.05) |
|  | Rural residence | 1.13 (0.71; 1.82) | 0.92 (0.56; 1.52) | 0.70 (0.44; 1.10) | 1.75 (0.95; 3.25) | 0.81 (0.42; 1.56) | 1.85 (0.53; 6.43) |
|  | Female | 0.91 (0.66; 1.26) | 1.13 (0.78; 1.64) | 1.30 (0.98; 1.72) | 0.71 (0.48; 1.03) | 1.36 (0.89; 2.06) | 0.77 (0.35; 1.66) |
|  | Household size >5 | 1.18 (0.84; 1.66) | 0.85 (0.57; 1.26) | 0.84 (0.62; 1.13) | 1.03 (0.68; 1.56) | 0.90 (0.58; 1.42) | 3.02 (1.19; 7.68) |
| Vietnam YC | Wealth Q1 | 1 (ref) | 1 (ref) | 1 (ref) | 1 (ref) | 1 (ref) | 1 (ref) |
|  | Wealth Q2 | 0.45 (0.35; 0.58) | 1.24 (0.94; 1.65) | 0.69 (0.46; 1.04) | 0.62 (0.31; 1.23) | 0.30 (0.11; 0.81) | NA |
|  | Wealth Q3 | 0.55 (0.43; 0.70) | 1.86 (1.37; 2.52) | 1.14 (0.79; 1.63) | 0.62 (0.32; 1.18) | 0.83 (0.4; 1.72) | NA |
|  | Wealth Q4 | 0.36 (0.26; 0.50) | 1.89 (1.23; 2.91) | 1.88 (1.29; 2.74) | 0.59 (0.30; 1.16) | 0.52 (0.19; 1.43) | NA |
|  | Rural residence | 1.71 (1.21; 2.40) | 0.67 (0.42; 1.05) | 0.35 (0.26; 0.46) | 1.56 (0.95; 2.55) | 1.10 (0.41; 2.9) | NA |
|  | Female | 0.91 (0.75; 1.10) | 1.14 (0.91; 1.42) | 0.58 (0.46; 0.73) | 1.47 (1.00; 2.15) | 0.72 (0.39; 1.31) | NA |
|  | Household size >5 | 1.91 (1.54; 2.38) | 0.74 (0.56; 0.98) | 1.01 (0.75; 1.34) | 0.91 (0.57; 1.46) | 1.27 (0.62; 2.59) | NA |
| Vietnam OC | Wealth Q1 | 1 (ref) | 1 (ref) | 1 (ref) | 1 (ref) | 1 (ref) | 1 (ref) |
|  | Wealth Q2 | 0.90 (0.60; 1.36) | 1.21 (0.82; 1.77) | 0.46 (0.20; 1.03) | 0.18 (0.04; 0.91) | 0.17 (0.02; 1.45) | NA |
|  | Wealth Q3 | 0.74 (0.49; 1.12) | 1.62 (1.07; 2.45) | 0.71 (0.35; 1.43) | 0.32 (0.09; 1.17) | 0.86 (0.25; 2.94) | NA |
|  | Wealth Q4 | 0.69 (0.43; 1.11) | 1.25 (0.74; 2.08) | 1.40 (0.72; 2.71) | 0.32 (0.09; 1.12) | 0.73 (0.20; 2.70) | NA |
|  | Rural residence | 1.44 (0.96; 2.16) | 0.91 (0.59; 1.42) | 0.37 (0.22; 0.63) | 0.75 (0.29; 1.98) | 0.30 (0.11; 0.84) | NA |
|  | Female | 1.17 (0.87; 1.58) | 2.05 (1.52; 2.77) | 0.75 (0.48; 1.18) | 1.5 (0.65; 3.51) | 1.43 (0.57; 3.58) | NA |
|  | Household size >5 | 1.87 (1.32; 2.66) | 0.80 (0.56; 1.16) | 0.99 (0.56; 1.77) | 3.99 (1.09; 14.54) | 0.49 (0.11; 2.14) | NA |

*Abbreviations:* YC, Younger Cohort; OC, Older Cohort; CSO, concurrent stunting and overweight.

*Note:* Wealth levels indicate quartiles of a wealth index (Q1 = poorest). OR indicate the odds of transitioning away compared to the odds of remaining in the respective state. Wealth is coded into quartiles and lowest quartiles (poorest) serve as reference. NA indicates that no OR could be calculated due to small numbers of individuals transitioning between the respective state.

# **Figure S1:** Marginal distribution probabilities of normal, stunted, overweight and concurrently stunted and overweight (CSO) for female and male children aged 1 to 15 (younger cohort (YC)).

# **Figure S2:** Transition probabilities between normal, stunted, overweight, concurrent stunting and overweight (CSO) state over time for females and males of the Younger Cohort (YC). Initial states are indicated in the columns and end states are indicated by colours.

**Figure S3:** Odds Ratios and 95% CI showing the relative difference for transitions into and out of stunting (A), overweight (B), and concurrent stunting and overweight (CSO, C) by sex, household size, residence, wealth index quartile, and maternal education from adjusted two-state models. No estimates are shown for transitions away from CSO due to small sample size.

*Note:* Wealth levels indicate quartiles of a wealth index (Q1 = poorest). This model is run with a sub-sample of the data with complete data on maternal education. Sample size for this model is smaller than for adjusted two-state models the presented in the main.
